# Piperazine- and Piperidine-Containing Thiazolo[5,4-*d*]pyrimidine Derivatives as New Potent and Selective Adenosine A_2A_ Receptor Inverse Agonists

**DOI:** 10.3390/ph13080161

**Published:** 2020-07-24

**Authors:** Flavia Varano, Daniela Catarzi, Erica Vigiani, Fabrizio Vincenzi, Silvia Pasquini, Katia Varani, Vittoria Colotta

**Affiliations:** 1 Dipartimento di Neuroscienze, Psicologia, Area del Farmaco e Salute del Bambino, Sezione di Farmaceutica e Nutraceutica, Universita’degli Studi di Firenze, Via Ugo Schiff 6, 50019 Sesto Fiorentino (FI), Italy; daniela.catarzi@unifi.it (D.C.); erica.vigiani@unifi.it (E.V.); vittoria.colotta@unifi.it (V.C.); 2 Dipartimento di Morfologia, Chirurgia e Medicina Sperimentale, Università degli Studi di Ferrara, Via Fossato di Mortara 17-19, 44121 Ferrara, Italy; fabrizio.vincenzi@unife.it (F.V.); silvia.pasquini@unife.it (S.P.); katia.varani@unife.it (K.V.)

**Keywords:** G protein-coupled receptors, adenosine receptors, adenosine A_2A_ receptor ligands, thiazolo[5,4-*d*]pyrimidines

## Abstract

The therapeutic use of A_2A_ adenosine receptor (AR) antagonists for the treatment of neurodegenerative disorders, such as Parkinson and Alzheimer diseases, is a very promising approach. Moreover, the potential therapeutic role of A_2A_ AR antagonists to avoid both immunoescaping of tumor cells and tumor development is well documented. Herein, we report on the synthesis and biological evaluation of a new set of piperazine- and piperidine- containing 7-amino-2-(furan-2-yl)thiazolo[5,4-*d*]pyrimidine derivatives designed as human A_2A_ AR antagonists/inverse agonists. Binding and potency data indicated that a good number of potent and selective hA_2A_ AR inverse agonists were found. Amongst them, the 2-(furan-2-yl)-*N*^5^-(2-(4-phenylpiperazin-1-yl)ethyl)thiazolo[5,4-*d*]pyrimidine-5,7-diamine **11** exhibited the highest A_2A_ AR binding affinity (K_i_ = 8.62 nM) as well as inverse agonist potency (IC_50_ = 7.42 nM). In addition, bioinformatics prediction using the web tool SwissADME revealed that **8**, **11**, and **19** possessed good drug-likeness profiles.

## 1. Introduction

**Figure 1:**
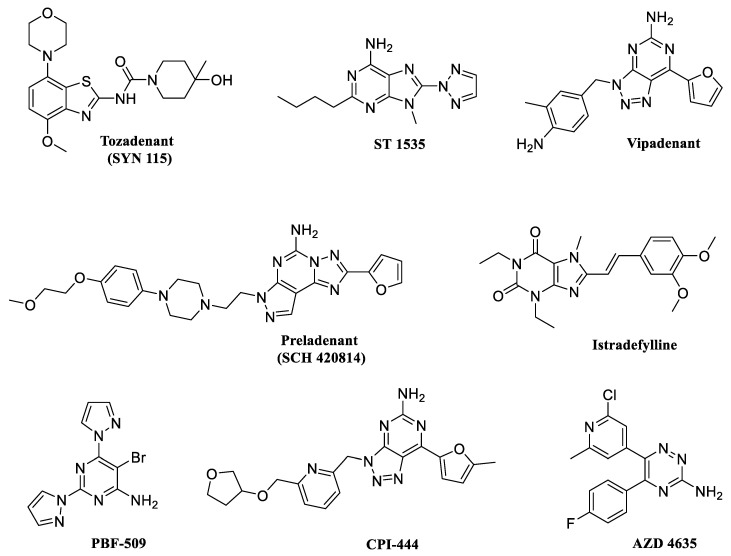
A_2A_ AR antagonists progressed into clinical testing for the treatment of Parkinson’s disease (PD) and cancer.

Adenosine is an endogenous purinergic nucleoside which interferes in many physiological states related to cardiovascular, immune, and neurological functions. Extracellular adenosine acts via four distinct G protein-coupled membrane receptors, namely A_1_, A_2A_, A_2B_, and A_3_ adenosine receptors (ARs). The A_1_ and A_3_ receptors are principally coupled to G_i/o_ proteins thus inducing an inhibitory effect on adenylyl cyclase and reducing cAMP production, while the A_2A_ and A_2B_ receptors stimulate the production of cAMP via G_s_ proteins [1]. ARs are distributed all over in the body and elevated adenosine levels and/or upregulation of ARs have been detected in many pathological conditions [2]. The A_2A_ AR is located both peripherally and centrally, with the highest expression levels in the striatum, olfactory tubercle, and the immune system. The A_2A_ AR is a very promising target in the field of neurodegenerative pathologies, mainly Parkinson’s (PD) and Alzheimer’s (AD) diseases [3,4,5]. Several A_2A_ AR antagonists have demonstrated to improve PD motor dysfunctions in various preclinical animal models as well as in clinical studies [5]. Furthermore, neuroprotective functions were associated with the use of A_2A_ AR antagonists thus suggesting that they may delay the onset and progression of PD [5]. The A_2A_ AR antagonists, such as Tozadenant (SYN 115) [6], ST 1535 [7], Vipadenant [8], Preladenant (SCH 420814) [9], and Istradefylline [10], have been clinically investigated showing potential effect in the treatment of PD. In particular, Istradefylline received marketing approval in Japan in 2013 as NOURIAST^®^ (Figure 1) and in 2019 was approved by the US Food and Drug Administration (FDA) for PD [11]. In the case of AD, it is well established that A_2A_ AR antagonists prevent amyloid beta toxicity accompanied by improvement of spatial memory [12].

Recently a large amount of research focused on the A_2A_ AR as a new target for cancer immunotherapy [13,14]. In fact, the A_2A_ AR represents an important immune checkpoint for T cells and NK cells and its activation induces suppression of immune cells response. Considering the increased A_2A_ AR expression in activated tumor infiltrating T cells, it is thus clear that this mechanism is important to favor tumor escape [15]. Moreover, A_2A_ AR is expressed also in tumor cells and its stimulation induces and increases cell proliferation, chemotaxis and migration, thus favoring tumor growth and metastasis [16]. The potential therapeutic role of A_2A_ AR antagonists to avoid immunoescaping of tumor cells and tumor development is evident. Indeed, four A_2A_ AR antagonists, including Preladenant [17], PBF-509 [18], CPI-444 [19], and AZD4635 [20] have entered clinical development as anticancer drugs alone and in combination with other agents (Figure 1).

Our group previously synthesized some potent human (h) A_2A_ AR antagonists/inverse agonists belonging to different chemical classes [21,22,23,24,25,26,27,28,29,30,31]. Among these, the thiazolo[5,4-*d*]pyrimidine one (TP series) has been deeply investigated allowing us to delineate comprehensive structure activity relationships [21,25,26,27,31]. This was possible because the central thiazolopyrimidine scaffold can be easily decorated by at least three different substituents at positions 2, 5, and 7, to explore diverse sites of interaction. To obtain potent and selective hA_2A_ AR antagonists/inverse agonists, the thiazolopyrimidine core must exhibit an exocyclic amine group at position 7 and a furan-2-yl moiety at position 2. In contrast, substituents endowed with variable properties, such as the steric hindrance, seems to be tolerated at position 5. In fact, good to high A_2A_ AR affinity was observed when an (hetero)aryl or alkyl residue was attached by diverse linkers at position 5 of the thiazolopyrimidine scaffold [21,25,26,27]. In particular, in a recent paper by us some interesting results were obtained when the linker was a piperazine moiety directly attached to the bicyclic core or spaced by an ethylamino chain [31]. It has to be noted that piperazine derivatives are reported to elicit a broad spectrum of pharmacological activities. In fact, this heterocycle is present in many well-known drugs belonging to diverse pharmacological classes [32].

**Figure 2:**
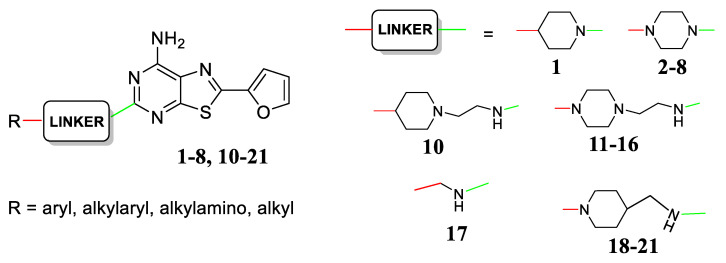
General structure of the designed 7-amino-2-(furan-2-yl)-thiazolo[5,4-*d*]pyrimidines.

Thus, to further investigate the structure-activity relationships of the 7-amino-2-(furan-2-yl)-thiazolo[5,4-*d*]pyrimidines as A_2A_ AR antagonists/inverse agonists, in the present paper we describe the synthesis of the new derivatives **1**–**8**, **10**–**21** (Figure 2) bearing at position 5 a piperidine or a piperazine moiety directly attached to the bicyclic core (**1**, and **2**–**8**, respectively) or spaced by an ethylamino chain (**10** and **11**–**16**, respectively). Moreover, a little set of compounds bearing at position 5 a methylamino (**17**) or a methylaminopiperidine chain (**18**–**21**) is reported.

## 2. Results

### 2.1. Chemistry

**Scheme 1:**
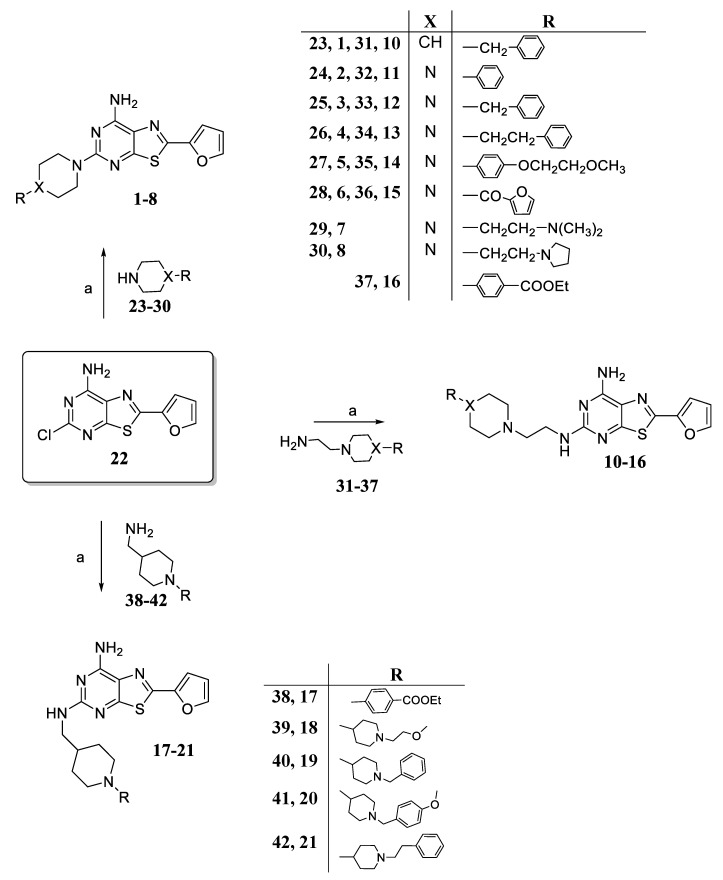
Reagents and conditions. (a) n-BuOH, 200 °C MW, 20 min.

Compounds **1**–**8**, **10**–**21** were prepared following a common procedure that first involved the obtainment of the 7-amino-5-chloro-2-(furan-2-yl)-thiazolo[5,4-*d*]pyrimidine **22** and of the appropriate amine tails **23**–**42**. Then, the two building blocks were reacted together to provide the desired compounds (Scheme 1).

**Scheme 2:**
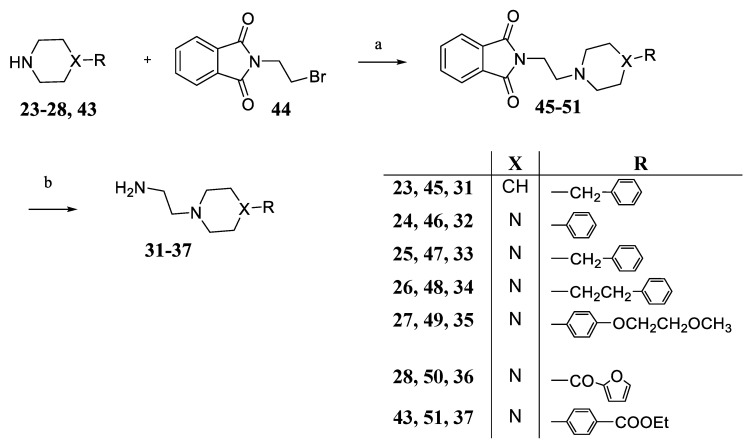
Reagents and conditions. (a) For **45**–**50**: CH_3_CN, K_2_CO_3_, reflux, 14 h; for **51**: CH_3_CN, Et_3_N, reflux, 24 h; (b) MeOH, NH_2_NH_2_·H_2_O, reflux, 2 h.

The 7-amino-5-chloro-thiazolo[5,4-*d*]pyrimidine **22** was prepared as previously described [21]. The reaction of the latter with an excess of the proper amine **23**–**42**, under microwave irradiation, delivered the target compounds **1**–**8**, **10**–**21**. The amines **23**–**24**, **26**, **28**–**30** were commercial, while **25**, **27,** and **38** were prepared according to the literature [33,34,35]. The ethylamine derivatives **31**–**37 [36,37]** were synthesized as outlined in Scheme 2.

Briefly, 4-benzylpiperidine **23** and the *N*^1^-substituted piperazines **24**–**28**, **43 [38]** were alkylated in standard conditions with *N*-(2-bromoethyl)phalimide **44** to achieve the *N*-(2-ethylsubstituted)phtalimide derivatives **45**–**51.** Removal of the phthaloyl group of the latters by hydrazinolysis produced compounds **31**–**37.**

**Scheme 3:**
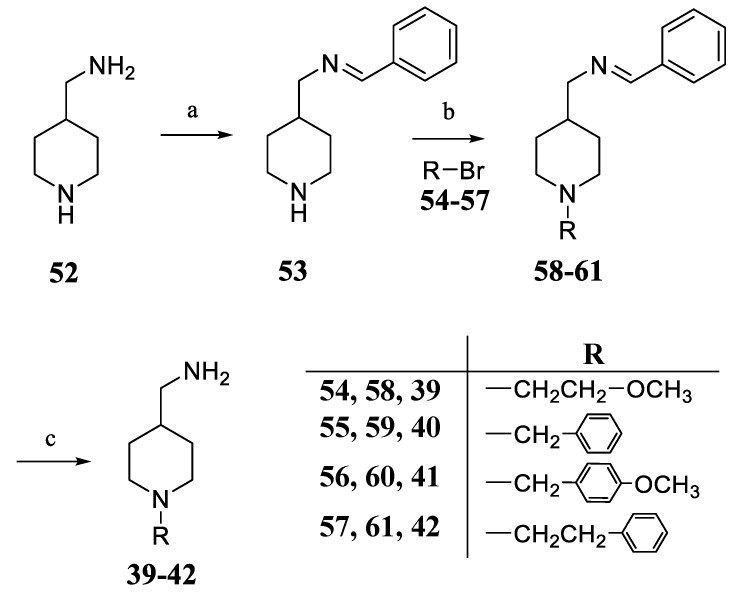
Reagents and conditions. (a) EtOH, benzaldehyde, reflux, 24 h; (b) CH_3_COCH_3_, K_2_CO_3_, rt, 12 h; (c) CH_2_Cl_2_/H_2_O, oxalic acid, reflux, 3 h.

Finally, the 4-aminomethyl-piperidine derivatives **39**–**42 [39,40]** were prepared starting from the commercial 4-aminomethylpiperidine **52**, following the reported procedure (Scheme 3) [31]. The reaction of the latter with benzaldehyde in absolute ethanol gave the imino derivative **53 [39]** which was then reacted with the proper alkyl(aryl)halide **54**–**57** to furnish, in satisfactory yields, the corresponding N-substituted piperidine derivatives **58**–**61 [39,40]**. Acidic hydrolysis of the protecting imino group of the latter gave the desired **39**–**42**.

### 2.2. Pharmacological Assays

Binding affinities of compounds **1**–**8, 10**–**21** for the hA_1_, hA_2A_, and hA_3_ AR subtypes, expressed in Chinese Hamster Ovary (CHO) cells, were determined in radioligand competition experiments. In the binding affinity assays, the competition of ligands for specific binding of [^3^H]DPCPX, [^3^H]ZM241385, and [^125^I]AB-MECA, respectively was measured to hA_1_, hA_2A_, and hA_3_ ARs. Activities of compounds **1**–**8**, **10**–**21** at the hA_2B_ AR subtype was determined by measuring the inhibition of NECA stimulated adenylyl cyclase activity in CHO cells expressing the hA_2B_ receptor. Compounds **8**, **11**, **14**–**15**, and **19**, the best in terms of hA_2A_ AR affinity and selectivity, were also evaluated for their functional behavior. Hence, compounds were tested to assess their ability to modulate cAMP production in hA_2A_ CHO cells. All pharmacological data are reported in Table 1 and Table 2 together with those of the reference compound ZM 241,385 [41].

## 3. Discussion

### 3.1. Structure-Activity Relationships

Binding and potency data of the newly synthesized compounds **1**–**8**, **10**–**21,** and of the previously reported derivative **9 [31]** are summarized in Table 1.

Most of the tested compounds (**2**–**3**, **6**–**8**, **10**–**15**, **19**–**20**) displayed high to good affinity for the hA_2A_ AR (8.62 nM < K_i_ < 187 nM). Instead, no significant affinity was detected for the off-target hARs with the exception of that of compounds **3**–**5**, **11**, **20** which bind the hA_1_ (**3**, **11**, **20**) and the hA_3_ subtypes (**4**–**5**) with good affinities.

Compounds **1**–**9** bear a piperidine (**1**) or a piperazine (**2**–**9**) substituted ring directly linked to the bicyclic thiazolopyrimidine core. Comparison of the hA_2A_ AR binding activity of the piperidine substituted **1** (K_i_ = 594 nM) and of its corresponding piperazine analogue **3** (K_i_ = 58 nM), both bearing an appended benzyl group, indicates that the piperazine linker is preferred. Analyzing the effect of different substituents on the piperazine ring, the data indicate that while an appended phenyl (**2**) or benzyl residue (**3**) was equally tolerated, a longer phenylethyl group (**4**) or a para-substituent (OCH_2_CH_2_OCH_3_, COOEt) on the phenyl ring of **2** (compounds **5** and **9,** respectively), produced a drop in the binding activity. Introduction of a furan-2-yl methanone residue on the piperazine ring gave compound **6** which shows good hA_2A_ AR affinity even if lower than that of **2** and **3**. In contrast, the presence of an ethylamine chain yielded derivatives **7**–**8** endowed with higher hA_2A_ AR affinity than that of **2** and **3**. Moreover, the (pyrrolidin-1-yl)ethyl derivative **8** is also highly selective toward this receptor subtype.

With respect to derivatives **1**–**6** and **9**, the piperidine or piperazine residue at position 5 of compounds **10**–**16** was shifted from the thiazolopyrimidine core by an ethylamino linker thus increasing chain flexibility. In general this structural change leads to an improved binding affinity with only two exceptions. In fact, while derivative **12** is slightly less active than its homologue **3,** the ethylbenzoate derivative **16** is equiactive to **9**. Among the herein reported compounds, the phenylpiperazine derivative **11** possesses the highest hA_2A_ AR affinity displaying a K_i_ value of 8.6 nM. Compared to the latter, the (furan-2-yl)methanonepiperazine derivative **15** shows a similar binding activity (K_i_ = 10.8 nM) but is more selective toward the hA_2A_ AR. Compound **14**, characterized by the same side chain of Preladenant**,** possesses high hA_2A_ AR affinity (K_i_ = 18.3 nM) similar to that of **11** and **15**, and is also highly selective.

Finally, the binding results of the last set of compounds (**17**–**21**), all characterized by an aminomethyl linker between the bicyclic core and the ethylbenzoate (**17**) or the substituted piperidine residue (**18**–**21**), indicate that only in one case, i.e., the benzyl piperidine derivative **19**, a high affinity (K_i_ = 15.2 nM) and a good selectivity toward the A_2A_ subtype is reached.

Selected compounds **8**, **11**, **14**–**15**, and **19**, the best in terms of hA_2A_ AR affinity and selectivity, were also evaluated in functional assays to assess their ability to modulate cAMP production in hA_2A_ CHO cells (Table 2, Figure S1). All the tested compounds behaved as inverse agonists since they were able to inhibit basal cAMP accumulation. In particular, according to their nanomolar hA_2A_ AR affinities, compounds **8**, **11**, **14**–**15**, and **19** show IC_50_ values spanning from 15.2 to 7.42 nM and also in this assay derivative **11** is the most active.

### 3.2. In Silico ADME Prediction

Compounds **8**, **11**, **14**–**15**, and **19** were also evaluated in silico to test their “drug-likeness” profiles on the basis of the absorption, distribution, metabolism, and excretion (ADME) properties. Calculations were performed by the SwissADME web service (http://www.swissadme.ch developed by the Molecular Modeling Group of the Swiss Institute of Bioinformatics) that gives free access to a pool of fast yet robust predictive models for small molecules pharmacokinetic properties [42]. The data evaluated for the selected compounds are summarized in the Supplementary Materials (Table S1).

Investigated molecules possessed several favorable ADME properties. All compounds obeyed the Lipinsky’s rule of five indicating drug-likeness. Moreover, they possessed good probability to have at least 10% oral bioavailability in rat or measurable Caco-2 permeability. SwissADME returns warnings if the molecule under evaluation contains fragments that could yield a false positive biological output (PAINS Pan Assay Interference Structures). Compounds **8**, **11**, **15**, and **19** had no PAINS alerts, while **14** showed one alert. The topological surface area (TPSA) measures the drug ability to permeate cells. Compounds **8**, **11**, and **19** showed similar TPSA values less than 140 Å^2^ suggesting that they could permeate cell membranes. The Consesus log P_o/w_ (octanol/water partition coefficient) values indicated rather a reasonable absorption (1.71 < Consensus log P_o/w_ < 3.34), while the log S values defined moderate solubility in the body.

**Figure 3:**
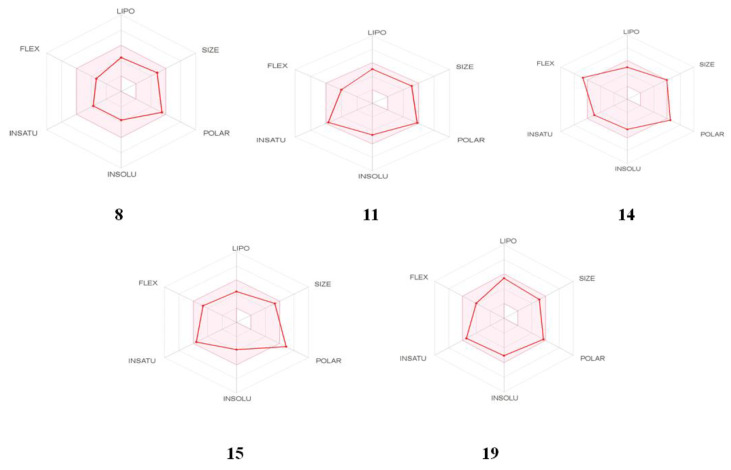
Bioavailability radars for the analyzed compounds, from the SwissADME web tool. LIPO = lipophilicity (XLOGP3 between −0.7 and 5.0); SIZE (molecular weight between 150 and 500 g/mol); POLAR = polarity (TPSA between 20 and 130 Å^2^); INSOLU = solubility (log S not higher than 6); INSATU = saturation (fraction of carbons in the sp^3^ hybridization not less than 0.25); FLEX = flexibility (no more than nine rotatable bonds).

The bioavailability radars (Figure 3) are the drug-likeness graphs of analyzed compounds presented in the form of a hexagon with each of the vertices representing a parameter (lipophilicity, size, polarity, solubility, flexibility, and saturation) that define a bioavailable drug. The pink region is the suitable physicochemical space for oral bioavailability. The radar plot of the molecule, represented by the red distorted hexagon, has to fall entirely in the pink area to be considered drug-like. From the graphs in Figure 3, it was found that while compounds **8**, **11**, and **19** were orally bioavailable, compounds **14** and **15** were not, because of being too polar and **14** also too flexible.

**Figure 4:**
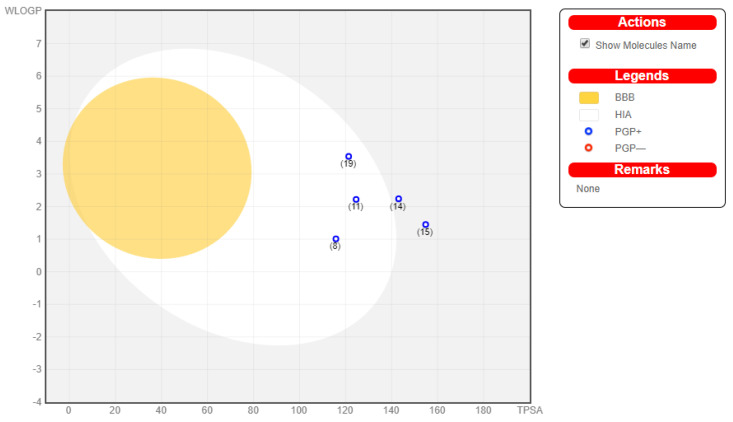
Predicted BOILED-Egg diagram of the analyzed compounds, from the SwissADME web tool.

Finally, the BOILED-egg (Brain Or IntestinaL EstimateD) method (Figure 4) allows predicting simultaneously two keys in vivo ADME parameters, i.e., the passive gastrointestinal absorption (HIA) and brain access (BBB) [43]. While all studied compounds had no BBB permeability (none in the yellow region), compounds **8**, **11**, and **19** exert high HIA (in the white region) and compounds **14** and **15** were not permeable (in the grey region). Moreover, they all were predicted as actively effluxed by Pgp (blue dots = PGP+).

## 4. Materials and Methods

### 4.1. Chemistry

#### 4.1.1. General Methods

The microwave-assisted syntheses were performed using an Initiator EXP Microwave Biotage instrument (frequency of irradiation: 2.45 GHz). Analytical silica gel plates (Merck F254, Kenilworth, NJ, USA), preparative silica gel plates (Merck F254, 2 mm), and silica gel 60 (Merck, 70–230 mesh) were used for analytical and preparative TLC, and for column chromatography, respectively. All melting points were determined on a Gallenkamp melting point apparatus and are uncorrected. Elemental analyses were performed with a FlashE1112 Thermofinnigan elemental analyzer for C, H, N and the results were within ±0.4% of the theoretical values. All final compounds revealed a purity not less than 95%. Compounds were named following IUPAC rules as applied by ChemDrawUltra 9.0. The IR spectra were recorded with a Perkin-Elmer Spectrum RX I spectrometer in Nujol mulls and are expressed in cm^−1^. NMR spectra were recorded on a Bruker Avance 400 spectrometer (400 MHz for ^1^H-NMR and 100 MHz for ^13^C-NMR). The chemical shifts are reported in δ (ppm) and are relative to the central peak of the solvent which was CDCl_3_ or DMSOd_6_. The following abbreviations are used: s: Singlet, d: Doublet, t: Triplet, m: Multiplet, br: Broad, and ar: Aromatic protons.

#### 4.1.2. General Procedure for the Synthesis of **1**–**8**, **10**–**21**

The proper amine **23**–**42** (3 mmol) was added to a solution of the 5-chloro-2-(furan-2-yl)thiazolo[5,4-*d*]pyrimidin-7-amine derivative **22** [21] (1 mmol) in n-BuOH (2 mL). The reaction mixture was microwave irradiated at 200 °C for 20 min, then cooled at room temperature and basified with an aqueous KOH solution (50%). Addition of water afforded a solid which was collected by filtration and washed with Et_2_O. The crude material was purified by crystallization or by chromatography.

5-(4-Benzylpiperidin-1-yl)-2-(furan-2yl)thiazolo[5,4-*d*]pyrimidin-7-amine (**1**). Yield 51%. Mp: 197–199 °C (acetonitrile). ^1^H-NMR (DMSO-*d*_6_): 1.06–1.15 (m, 2H), 1.60 (d, 2H, *J* = 12 Hz), 1.77 (br s, 1H), 2.77 (t, 2H, *J* = 13 Hz), 4.64 (d, 2H, *J* = 12 Hz), 6.72–6.73 (m, 1H, ar), 7.04–7.05 (m, 1H, ar), 7.17–7.30 (m, 7H, 5ar + NH_2_), 7.90 (s, 1H, ar). Anal. calcd. for (C_21_H_21_N_5_OS): C, 64.43%; H, 5.41%; N, 17.89%. Anal. found: C, 64.55%; H 5.77%; N 18.13%.

2-(Furan-2-yl)-5-(4-phenylpiperazin-1-yl)thiazolo[5,4-*d*]pyrimidin-7-amine (**2**). Yield 30%. Mp: 219–221 °C (ethanol). ^1^H-NMR (CDCl_3_): 3.27 (t, 4H, *J* = 5.1 Hz), 4.02 (t, 4H, *J* = 5.1 Hz), 5.49 (br s, 2H, NH_2_), 6.57–6.58 (m, 1H, ar), 6.91 (t, 1H, ar, *J* = 7.3 Hz), 6.99–7.02 (m, 3H, ar), 7.29–7.33 (m, 2H, ar), 7.57–7.58 (m, 1H, ar). ^13^C-NMR (DMSO-*d*_6_): 164.99, 159.26, 157.29, 151.56, 148.58, 146.30, 129.42, 125.09, 119.63, 116.32, 113.20, 110.26, 48.87, 44.20. IR: 3172, 3213, 3300, 3392. Anal. calcd. for (C_19_H_18_N_6_OS): C, 60.30%; H, 4.79%; N, 22.21%. Anal. found: C, 60.43%; H, 5.08%; N, 22.49%.

5-(4-Benzylpiperazin-1-yl)-2-(furan-2-yl)thiazolo[5,4-*d*]pyrimidin-7-amine (**3**). The crude product was purified by column chromatography, eluting system ethyl acetate/cyclohexane 7/3. Yield 40%. Mp: 181–183 °C. ^1^H-NMR (DMSO-*d*_6_): 2.34–2.44 (m, 4H), 3.51 (s, 2H), 3.73–3.75 (m, 4H), 6.72–6.73 (m, 1H, ar), 7.06–7.08 (m, 1H, ar), 7.23–7.34 (m, 7H, 5 ar + NH_2_), 7.90–7.91 (m, 1H, ar).^13^C-NMR (DMSO-*d*_6_): 164.97, 159.24, 157.21, 148.58, 146.12, 138.54, 124.44, 129.24, 128.67, 128.58, 127.43, 127.31, 113.19, 110.16, 62.59, 53.01, 44.27. IR: 3149, 3172, 3211, 3304. Anal. calcd. for (C_20_H_20_N_6_OS): C, 61.21%; H, 5.14%; N, 21.41%. Anal. found: C, 61.48%; H, 5.51%; N, 21.74%.

2-(Furan-2-yl)-5-(4-phenethylpiperazin-1-yl)thiazolo[5,4-*d*]pyrimidin-7-amine (**4**). The crude product was purified by column chromatography, eluting system ethyl acetate/cyclohexane 7/3. Yield 30%. Mp: 203–204 °C (ethanol). ^1^H-NMR (DMSO-*d*_6_): 2.52–2.57 (m, 6H), 2.75–2.79 (m, 2H), 3.72–3.77 (s, 4H), 6.72–6.73 (m, 1H, ar), 7.06–7.07 (m, 1H, ar), 7.17–7.31 (m, 7H, 5 ar + NH_2_), 7.91–7.92 (s, 1H, ar).^13^C-NMR (DMSO-*d*_6_): 164.97, 159.26, 157.20, 148.56, 146.09, 140.86, 129.11, 128.70, 126.30, 124.93, 113.22, 110.18, 60.25, 53.06, 44.30, 33.17. IR: 3280, 3421. Anal. calcd. for (C_21_H_22_N_6_OS): C, 62.05%; H, 5.46%; N, 20.67%. Anal. found: C, 61.98%; H, 5.54%; N, 21.03%.

2-(Furan-2-yl)-5-(4-(4-(2-methoxyethoxy)phenyl)piperazin-1-yl)thiazolo[5,4-*d*]pyrimidin-7-amine (**5**). Yield 50%. Mp: 181–183 °C (methanol). ^1^H-NMR (DMSO-*d*_6_): 3.05–3.08 (m, 4H). 3.29 (s, 3H), 3.62 (t, 2H, *J* = 5.0 Hz), 3.86–3.89 (m, 4H), 4.01 (t, 2H, *J* = 5 Hz), 6.72–6.73 (m, 1H, ar), 6.84 (d, 2H, ar, *J* = 9.0 Hz), 6.94 (d, 2H, ar, *J* = 9.0 Hz), 7.07–7.08 (m, 1H, ar), 7.32 (br s, 2H, NH_2_), 7.91 (s, 1H, ar). Anal. calcd. for (C_22_H_24_N_6_O_3_S): C, 58.39%; H, 5.35%; N, 18.57%. Anal. found: C, 58.68%; H, 5.58%; N, 18.79%.

(4-(7-Amino-2-(furan-2-yl)thiazolo[5,4-*d*]pyrimidin-5-yl)piperazin-1-yl)(furan-2-yl)methanone (**6**). The crude product was purified by column chromatography, eluting system ethyl acetate/cyclohexane 7/3. Yield 25%. Mp: 227–229 °C (tetrahydrofuran/water). ^1^H-NMR (DMSO-*d*_6_): 3.74–3.81 (m, 8H), 6.65–6.66 (m, 1H, ar), 6.74–6.75 (m, 1H, ar), 7.04–7.05 (m, 1H, ar), 7.08–7.09 (m, 1H, ar), 7.39 (s, 2H, NH_2_), 7.87 (s, 1H, ar), 7.92 (s, 1H, ar). ^13^C-NMR (CDCl_3_): 165.47, 159.31, 158.89, 156.28, 148.70, 147.91, 144.07, 143.82, 125.30, 116.65, 112.33, 111.38, 110.07, 60.41, 44.38, 26.91, 21.07, 14.21. IR: 3429, 3307, 3209, 1620. Anal. calcd. for (C_18_H_16_N_6_O_3_S): C, 54.54%; H, 4.07%; N, 21.20%. Anal. found: C, 54.00%; H, 4.29%; N, 21.39%.

5-(4-(2-(Dimethylamino)ethyl)piperazin-1-yl)-2-(furan-2-yl)thiazolo[5,4-*d*]pyrimidin-7-amine (**7**). The product was purified by column chromatography, eluting system chloroform/methanol/ammonium hydroxide 8.5/1.5/0.15. Yield 38%. Mp: 183–186 °C. ^1^H-NMR (DMSO-*d*_6_): 2.15 (s, 6H), 2.36–2.44 (m, 8H), 3.69–3.71 (m, 4H), 6.72–6.73 (m, 1H, ar), 7.06–7.07 (m, 1H, ar), 7.26 (s, 2H, NH_2_), 7.90–7.91 (m, 1H, ar). Anal. calcd. for (C_17_H_23_N_7_OS): C, 54.67%; H, 6.21%; N, 26.25%. Anal. found: C, 55.01%; H, 6.54%; N, 26.39%.

2-(Furan-2-yl)-5-(4-(2-(pyrrolidin-1-yl)ethyl)piperazin-1-yl)thiazolo[5,4-*d*]pyrimidin-7-amine (**8**). The product was purified by column chromatography, eluting system chloroform/methanol/ammonium hydroxide 8.5/1.5/0.15. Yield 25%. Mp: 181–183 °C. ^1^H-NMR (DMSO-*d*_6_): 1.59–1.65 (m, 4H), 2.40–2.49 (m, 12H), 3.65–3.69 (m, 4H), 6.72–6.73 (m, 1H, ar), 7.06–7.07 (m, 1H, ar), 7.29 (s, 2H, NH_2_), 7.90–7.91 (m, 1H, ar). Anal. calcd. for (C_19_H_25_N_7_OS): C, 57.12%; H, 6.31%; N, 24.54%. Anal. found: C, 56.89%; H, 6.45%; N, 24.77%.

*N*^5^-(2-(4-Benzylpiperidin-1-yl)ethyl)-2-(furan-2-yl)thiazolo[5,4-*d*]pyrimidine-5,7-diamine (**10**). Yield 22%. Mp: 155-157 °C (ethyl acetate). ^1^H-NMR (CDCl_3_): 1.28–1.31 (m, 2H), 1.34–1.37 (m, 1H), 1.56–1.57 (m, 2H), 1.95 (t, 2H, *J* = 11.2 Hz), 2.55–2.57 (m, 4H), 2.91 (d, 2H, *J* = 11.2 Hz), 3.48–3.52 (m, 2H), 5.48 (br s, 2H, NH_2_), 5.58 (br s, 1H, NH), 6.57–6.58 (m, 1H, ar), 6.97–6.98 (m, 1H, ar), 7.15–7.32 (m, 5H, ar), 7.56 (s, 1H, ar). ^13^C-NMR (DMSO-*d*_6_): 165.01, 160.30, 157.45, 148.63, 140.86, 129.43, 128.56, 126.15, 113.15, 109.98, 57.77, 53.86, 42.87, 37.89, 32.26. IR: 3323, 3169, 3116. Anal. calcd. for (C_23_H_26_N_6_OS): C, 63.57%; H, 6.03%; N, 19.34%. Anal. found: C, 63.88%; H, 6.36%; N, 19.21%.

2-(Furan-2-yl)-*N*^5^-(2-(4-phenylpiperazin-1-yl)ethyl)thiazolo[5,4-*d*]pyrimidine-5,7-diamine (**11**). Yield 60%. Mp: 218–220 °C (2-methoxyethanol). ^1^H-NMR (CDCl_3_): 2.67–2.72 (m, 6H), 3.23–3.25 (m, 4H), 3.56–3.60 (m, 2H), 5.49 (br s, 2H, NH_2_), 5.58 (br s, 1H, NH), 6.57–6.58 (m, 1H, ar), 6.88 (t, 1H, ar, *J* = 7.2 Hz), 6.95–6.99 (m, 3H, ar), 7.27–7.31 (m, 2H, ar), 7.57 (s, 1H, ar). ^13^C-NMR (DMSO-*d*_6_): 160.36, 157.49, 151.54, 148.64, 129.36, 119.17, 115.77, 113.14, 110.01, 57.55, 53.25, 48.70, 38.83. IR: 3334, 3263, 3165, 3115. Anal. calcd. for (C_21_H_23_N_7_OS): C, 59.84%; H, 5.50%; N, 23.26%. Anal. found: C, 60.19%; H, 5.55%; N, 23.55%.

*N*^5^-(2-(4-Benzylpiperazin-1-yl)ethyl)-2-(furan-2-yl)thiazolo[5,4-*d*]pyrimidine-5,7-diamine (**12**). The product was purified by column chromatography, eluting system ethyl acetate/cyclohexane/methanol 6.5/2/1.5. Yield 45%. Mp: 133–135 °C. ^1^H-NMR (CDCl_3_): 2.54–2.60 (m, 10H), 3.50–3.54 (m, 4H), 5.53 (br s, 2H, NH_2_), 5.63 (br s, 1H, NH), 6.56–6.58 (m, 1H, ar), 6.97–6.98 (m, 1H, ar), 7.28–7.34 (m, 5H, ar), 7.56 (s, 1H, ar).^13^C-NMR (DMSO-*d*_6_): 160.29, 157.44, 148.61, 138.68, 133.04, 129.26, 127.31, 125.56, 113.12, 110.00, 62.54, 57.47, 53.24, 53.08. IR: 3331, 3265, 3174, 3082. Anal. calcd. for (C_22_H_25_N_7_OS): C, 60.67%; H, 5.79%; N, 22.51%. Anal. found: C, 60.49%; H, 6.09%; N, 22.43%.

2-(Furan-2-yl)-*N*^5^-(2-(4-phenethylpiperazin-1-yl)ethyl)thiazolo[5,4-*d*]pyrimidine-5,7-diamine (**13**). The product was purified by column chromatography, eluting system ethyl acetate/cyclohexane/methanol 6.5/2/1.5. Yield 37%. Mp: 151–153 °C. ^1^H-NMR (CDCl_3_): 2.61–2.66 (m, 12H), 2.82–2.86 (m, 2H), 3.48–3.57 (m, 2H), 5.51 (br s, 2H, NH_2_), 5.60 (br s, 1H, NH), 6.56–6.58 (m, 1H, ar), 6.98–6.99 (m, 1H, ar), 7.20–7.33 (m, 5H, ar), 7.56 (s, 1H, ar). ^13^C-NMR (DMSO-*d*_6_): 160.30, 157.46, 148.63, 140.95, 133.00, 129.07, 128.24, 125.60, 113.15, 109.99, 60.24, 57.50, 53.25, 53.21, 33.22. IR: 3325, 3259, 3184, 3105. Anal. calcd. for (C_23_H_27_N_7_OS): C, 61.45%; H, 6.05%; N, 21.81%. Anal. found: C, 61.63%; H, 6.34%; N, 22.11%.

2-(Furan-2-yl)-*N*^5^-(2-(4-(4-(2-methoxyethoxy)phenyl)piperazin-1-yl)ethyl)thiazolo[5,4-*d*]pyrimidine-5,7-diamine (**14**). Yield 25%. Mp: 203–204 °C (ethanol). ^1^H-NMR (CDCl_3_): 2.67–2.68 (m, 6H), 3.13–3.14 (m, 4H), 3.47 (s, 3H), 3.55–3.59 (m, 2H), 3.74–3.76 (m, 2H), 4.09–4.11 (m, 2H), 5.50 (br s, 2H*,* NH_2_), 5.57 (br s, 1H, NH), 6.57–6.58 (m, 1H, ar), 6.90 (br s, 4H, ar), 6.98–6.99 (m, 1H, ar), 7.56 (s, 1H, ar). ^13^C-NMR (CDCl_3_): 159.78, 156.65, 152.94, 148.76, 145.92, 143.96, 118.05, 115.41, 112.26, 109.88, 71.20, 67.75, 59.19, 56.85, 53.05, 50.51, 38.31. IR: 3325, 3263, 3167. Anal. calcd. for (C_24_H_29_N_7_O_3_S): C, 58.16%; H, 5.90%; N, 19.78%. Anal. found: C, 58.29%; H, 5.63%; N, 20.05%.

(4-(2-((7-Amino-2-(furan-2-yl)thiazolo[5,4-*d*]pyrimidin-5-yl)amino)ethyl)piperazin-1-yl)(furan-2-yl)methanone (**15**). The product was purified by column chromatography, eluting system ethyl acetate/cyclohexane/methanol 6/4/0.5. Yield 33%. Mp: 163–165 °C. ^1^H-NMR (CDCl_3_): 2.57–2.59 (m, 4H), 2.65 (t, 2H, *J* = 5.9 Hz), 3.54–3.58 (m, 2H), 3.85 (br s, 4H), 5.53–5.55 (m, 3H, NH + NH_2_), 6.49–6.50 (m, 1H, ar), 6.57–6.58 (m, 1H, ar), 6.98–6.99 (m, 1H, ar), 7.01–7.02 (m, 1H, ar), 7.50 (s, 1H, ar), 7.57 (s, 1H, ar). ^13^C-NMR (DMSO-*d*_6_): 164.97, 160.31, 158.70, 157.45, 148.61, 147.51, 145.07, 115.91, 113.15, 111.71, 110.00, 57.33, 53.27, 38.65. Anal. calcd. for (C_20_H_21_N_7_O_3_S): C, 54.66%; H, 4.82%; N, 22.31%. Anal. found: C, 54.94%; H, 5.18%; N, 22.67%.

Ethyl 4-(4-(2-((7-amino-2-(furan-2-yl)thiazolo[5,4-*d*]pyrimidin-5-yl)amino)ethyl)piperazin-1-yl) benzoate (**16**). The product was purified by column chromatography, eluting system ethyl acetate/cyclohexane/methanol 5/4/1. Yield 17%. Mp: 242–244 °C. ^1^H-NMR (CDCl_3_): 1.27 (t, 3H, *J* = 7.0 Hz), 2.59–2.72 (m, 6H), 3.33–3.38 (m, 4H), 3.57–3.58 (m, 2H), 4.35 (q, 2H, *J* = 7.0 Hz), 4.49 (br s, 1H, NH), 5.52 (br s, 2H, NH_2_), 6.57 (s, 1H, ar), 6.89 (d, 2H, ar, *J* = 8.7 Hz), 6.98–6.99 (m, 1H, ar), 7.57–7.59 (m, 1H, ar), 7.95 (d, 2H, ar, *J* = 8.7 Hz). Anal. calcd. for (C_24_H_27_N_7_O_3_S): C, 58.40%; H, 5.51%; N, 19.86%. Anal. found: C, 58.67%; H, 5.82%; N, 20.10%.

Ethyl 4-(((7-amino-2-(furan-2-yl)thiazolo[5,4-*d*]pyrimidin-5-yl)amino)methyl)benzoate (**17**). The product was purified by column chromatography, eluting system ethyl acetate/cyclohexane 4/6. Yield 36%. Mp: 218–220 °C. ^1^H-NMR (DMSO-*d*_6_): 1.31 (t, 3H, *J* = 7.3 Hz), 4.29 (q, 2H, *J* = 7.3 Hz), 4.53–4.61 (m, 2H), 6.70–6.71 (m, 1H, ar), 7.03–7.07 (m, 1H, ar), 7.20 (s, 2H, NH_2_), 7.41–7.50 (m, 3H, 2ar + NH), 7.86–7.94 (m, 3H, ar). ^13^C-NMR (DMSO-*d*_6_): 164.95, 159.22, 157.22, 148.55, 146.15, 143.28, 142.87, 124.95, 113.23, 110.21, 65.38, 61.86, 53.02, 44.45. Anal. calcd. for (C_19_H_17_N_5_O_3_S): C, 57.71%; H, 4.33%; N, 17.71%. Anal. found: C, 57.58%; H, 4.65%; N, 17.88%.

2-(Furan-2-yl)-*N*^5^-((1-(2-methoxyethyl)piperidin-4-yl)methyl)thiazolo[5,4-*d*]pyrimidine-5,7-diamine (**18**). The product was purified by column chromatography, eluting system chloroform/methanol 8/2. Yield 20%. Mp: 143–146 °C. ^1^H-NMR (CDCl_3_): 1.45–1.48 (m, 2H), 1.79–1.82 (m, 4H), 2.07–2.11 (m, 2H), 2.59–2.63 (m, 2H), 3.04–3.06 (m, 2H), 3.33–3.37 (m, 4H), 3.57 (t, 2H, *J* = 5.5 Hz), 5.03 (t, 1H, NH, *J* = 5.8Hz), 5.49 (br s, 2H, NH_2_), 6.56–6.58 (m, 1H, ar), 6.97–6.98 (m, 1H, ar), 7.56 (s, 1H, ar). ^13^C-NMR (DMSO-*d*_6_): 165.03, 160.63, 157.41, 148.67, 113.17, 109.96, 70.36, 58.46, 57.77, 53.95, 47.12, 35.85, 30.21. IR: 3315, 3261, 3178. Anal. calcd. for (C_18_H_24_N_6_O_2_S): C, 55.65%; H, 6.23%; N, 21.63%. Anal. found: C, 55.98%; H, 5.98%; N, 21.77%.

*N*^5^-((1-Benzylpiperidin-4-yl)methyl)- 2-(furan-2-yl)thiazolo[5,4-*d*]pyrimidine-5,7-diamine (**19**). Yield 17%. Mp: 188–190 °C (ethyl acetate). ^1^H-NMR (DMSO-*d*_6_): 1.12–1.20 (m, 2H), 1.50–1.55 (m, 1H), 1.64–1.67 (m, 2H), 1.87 (t, 2H, *J* = 10.7 Hz), 2.78 (d, 2H, *J* = 11.3 Hz), 3.15 (t, 2H, *J* = 6.2 Hz), 3.40 (s, 2H), 6.71–6.72 (m, 1H, ar), 6.87 (br s, 1H, NH), 7.03–7.04 (m, 1H, ar), 7.11 (br s, 2H, NH_2_), 7.21–7.33 (m, 5H, ar), 7.89 (s, 1H, ar). ^13^C-NMR (DMSO-*d*_6_): 160.62, 157.41, 148.67, 139.24, 129.15, 128.54, 127.19, 113.13, 109.92, 62.94, 53.51, 36.07, 30.37. IR: 3311, 3263, 3201. Anal. calcd. for (C_22_H_24_N_6_OS): C, 62.83%; H, 5.75%; N, 19.98%. Anal. found: C, 63.15%; H, 5.59%; N, 20.21%.

2-(Furan-2-yl)-*N*^5^-((1-(4-methoxybenzyl)piperidin-4-yl)methyl)thiazolo[5,4-*d*]pyrimidine-5,7-diamine (**20**). The product was purified by column chromatography, eluting system chloroform/methanol 8/2. Yield 15%. Mp: 182–183 °C. ^1^H-NMR (CDCl_3_): 1.34–1.40 (m, 2H), 1.61–1.78 (m, 3H), 1.96 (t, 2H, *J* = 11.0 Hz), 2.92 (d, 2H, *J* = 10.7 Hz), 3.34 (t, 2H, *J* = 6.1 Hz), 3.46 (s, 2H), 3.82 (s, 3H), 5.01 (br s, 1H, NH), 5.47 (br s, 2H, NH_2_), 6.57 (m, 1H, ar), 6.86 (d, 2H, *J* = 8.3 Hz), 6.96–6.97 (m, 1H, ar), 7.24 (d, 2H, ar, *J* = 8.3 Hz), 7.56 (s, 1H, ar). ^13^C-NMR (DMSO-*d*_6_): 160.62, 158.72, 157.51, 148.62, 130.94, 130.41, 113.94, 113.18, 109.99, 62.29, 55.49, 53.37, 47.14, 36.09, 30.30. IR: 3313, 3255, 3197. Anal. calcd. for (C_23_H_26_N_6_O_2_S): C, 61.31%; H, 5.82%; N, 18.65%. Anal. found: C, 61.49%; H, 5.75%; N, 18.78%.

2-(Furan-2-yl)-*N*^5^-((1-phenethylpiperidin-4-yl)methyl)thiazolo[5,4-*d*]pyrimidine-5,7-diamine (**21**). The product was purified by column chromatography, eluting system chloroform/methanol 8/2. Yield 27%. Mp: 174–176 °C. ^1^H-NMR (CDCl_3_): 1.40–1.43 (m, 2H), 1.82–1.85 (m, 3H), 2.06–2.11 (m, 2H), 2.59–2.62 (m, 2H), 2.83–2.84 (m, 2H), 3.06–3.11 (m, 2H), 3.36–3.39 (m, 2H), 5.03 (br s, 1H, NH), 5.47 (br s, 2H, NH_2_), 6.57–6.58 (m, 1H, ar), 6.98–6.99 (m, 1H, ar), 7.22–7.30 (m, 5H, ar), 7.56 (s, 1H, ar). ^13^C-NMR (DMSO-*d*_6_): 160.62, 157.41, 148.66, 141.05, 129.11, 128.67, 126.21, 113.13, 109.87, 60.59, 53.52, 47.18, 36.09, 33.35, 30.35. IR: 3311, 3267, 3197. Anal. calcd. for (C_23_H_26_N_6_OS): C, 63.57%; H, 6.03%; N, 19.34%. Anal. found: C, 63.72%; H, 6.39%; N, 19.51%.

#### 4.1.3. General Procedure for the Synthesis of **31**–**32**

In a 50 mL flask, equipped with a magnetic stirrer and reflux condenser, the proper phthalimide derivatives **45**–**46** (7 mmol), hydrazine hydrate (10 mmol), and methanol (50 mL) were added. The resulting mixture was refluxed for 2 h, cooled down to room temprature and concentrated under reduced pressure. The remaining residue was dissolved in a NaOH aqueous solution (1 M, 30 mL), washed with ethyl acetate (3 × 30 mL) dried over Na_2_SO_4_ and concentrated under reduced pressure. The oily residue without further purification was used in the following step.

2-(4-Benzylpiperidin-1-yl)ethan-1-amine (**31**). Yield 85%. ^1^H-NMR (CDCl_3_): 1.26–1.36 (m, 2H), 1.50–1.66 (m, 3H), 1.90 (t, 2H, *J* = 10.7 Hz), 2.39 (t, 2H, *J* = 6.3 Hz), 2.54–2.56 (m, 2H), 2.79 (t, 2H, *J* = 6.3 Hz), 2.88 (d, 2H, *J* = 11.6 Hz), 7.15–7.31 (m, 5H, ar).

2-(4-Phenylpiperazin-1-yl)ethan-1-amine (**32**) [36]. Yield 63%. ^1^H-NMR (CDCl_3_): 2.51 (t, 2H, *J* = 5.8 Hz), 2.63–2.66 (m, 4H), 2.86 (t, 2H, *J* = 5.8 Hz), 3.22–3.24 (m, 4H), 6.87 (t, 1H, ar, *J* = 7.2 Hz), 6.95 (d, 2H, ar, *J* = 8.1 Hz), 7.28 (t, 2H, ar, *J* = 7.9 Hz).

#### 4.1.4. General Procedure for the Synthesis of **33**–**36**

In a 50 mL flask, equipped with a magnetic stirrer and reflux condenser, the proper phthalimide derivatives **47**–**50** (7 mmol), hydrazine hydrate (10 mmol), and methanol (50 mL) were added. The resulting mixture was refluxed for 2 h, cooled down to room temprature, and concentrated under reduced pressure. The remaining residue was treated with diethyl ether and the solid was filtered and used without further purification in the following step.

2-(4-Benzylpiperazin-1-yl)ethan-1-amine (**33**) [37]. Yield 85%. ^1^H-NMR (CDCl_3_): 2.10 (br s, 4H), 2.34–2.56 (m, 6H), 2.82 (t, 2H, *J* = 6.0 Hz), 3.52 (s, 2H), 7.26–7.33 (m, 5H, ar).

2-(4-Phenethylpiperazin-1-yl)ethan-1-amine (**34**). Yield 90%. ^1^H-NMR (CDCl_3_): 1.73 (br s, 4H), 2.46 (t, 2H, *J* = 6.2 Hz), 2.56–2.65 (m, 8H), 2.81–2.85 (m, 4H), 7.20–7.32 (m, 5H, ar).

2-(4-(4-(2-Methoxyethoxy)phenyl)piperazin-1-yl)ethan-1-amine (**35**). Yield 30%. ^1^H-NMR (CDCl_3_): 2.51 (t, 2H, *J* = 6.1 Hz), 2.63–2.65 (m, 4H), 2.85 (t, 2H, *J* = 6.1 Hz), 3.11–3.13 (m, 4H), 3.46 (s, 3H), 3.74 (t, 2H, *J* = 4.7 Hz), 4.10 (t, 2H, *J* = 4.7 Hz), 6.87–6.92 (m, 4H, ar).

(4-(2-Aminoethyl)piperazin-1-yl)(furan-2-yl)methanone (**36**). Yield 60%. ^1^H-NMR (CDCl_3_): 2.47–2.54 (m, 6H), 2.83 (t, 2H, *J* = 6.1 Hz), 3.83 (br s, 4H), 6.49–6.50 (m, 1H, ar), 6.99–7.00 (m, 1H, ar), 7.49 (s, 1H, ar).

#### 4.1.5. Ethyl 4-(4-(2-aminoethyl)piperazin-1-yl)benzoate (**37**)

In a 50 mL flask, equipped with a magnetic stirrer and reflux condenser, the proper phthalimide derivatives **51** (7 mmol), hydrazine hydrate (8.4 mmol), and methanol (30 mL) were added. The resulting mixture was refluxed for 2 h, cooled down to room temprature and concentrated under reduced pressure. The remaining residue was treated with a solution of HCl 1 M and the solid residue was filtered. The acidic solution was alkalinized with Et_3_N and the obtained precipitate was filtered and used without further purification in the following step. Yield 50%. ^1^H-NMR (DMSO-*d*_6_): 1.29 (t, 3H, *J* = 7.1 Hz), 2.34 (t, 2H, *J* = 6.5 Hz), 2.48–2.60 (m, 4H), 2.65 (t, 2H, *J* = 6.5 Hz), 3.29–3.31 (m, 4H), 4.24 (q, 2H, *J* = 7.1 Hz), 6.97 (d, 2H, ar), 7.78 (d, 2H, ar).

#### 4.1.6. General Procedure for the Synthesis of **39**–**42**

To a solution of **58**–**61** (5.6 mmol) in dichloromethane (60 mL), oxalic acid (6.3 mmol) was added. The solution was diluted with water (30 mL) and refluxed under vigorous stirring for 3 h. After cooling, the aqueous layer was isolated, washed twice with dichloromethane (30 mL), added with a NaOH aqueous solution (1 M, pH 9–10), and extracted with chloroform (50 mL × 3). The organic layer was dried over Na_2_SO_4_, evaporated under reduced pressure, and the obtained oily residue was used as such in the next step.

(1-(2-Methoxyethyl)piperidin-4-yl)methanamine (**39**). Yield 60%. ^1^H-NMR (DMSO-*d*_6_): 1.01–1.11 (m, 3H), 1.60-1.63 (m, 2H), 1.86 (t, 2H, *J* = 10.7 Hz), 2.35–2.43 (m, 4H), 2.81–2.84 (m, 2H), 3.22 (s, 3H) 3.39 (t, 2H, *J* = 6.0 Hz).

(1-Benzylpiperidin-4-yl)methanamine (**40**) [39]. Yield 35%. ^1^H-NMR (DMSO-*d*_6_): 1.15–1.17 (m, 3H), 1.62–1.65 (m, 2H), 1.86 (t, 2H, *J* = 11.5 Hz), 2.38–2.40 (m, 2H), 2.78 (d, 2H, *J* = 11.5 Hz), 3.41 (s, 2H), 7.23–7.33 (m, 5H, ar).

(1-(4-Methoxybenzyl)piperidin-4-yl)methanamine (**41**). Yield 55%. ^1^H-NMR (CDCl_3_): 1.27–1.29 (m, 3H), 1.70–1.72 (m, 2H), 1.94 (t, 2H, *J* = 11.2 Hz), 2.58–2.59 (m, 2H), 2.92 (d, 2H, *J* = 11.6Hz), 3.46 (s, 2H), 3.82 (s, 3H), 6.87 (d, 2H, ar, *J* = 8.5 Hz), 7.24 (d, 2H, ar, *J* = 8.5 Hz).

(1-Phenethylpiperidin-4-yl)methanamine (**42**) [40]. Yield 63%. ^1^H-NMR (CDCl_3_): 1.27–1.31 (m, 3H), 1.75–1.78 (m, 2H), 2.03 (t, 2H, *J* = 10.7 Hz), 2.58–2.62 (m, 4H), 2.81–2.86 (m, 2H), 3.05 (d, 2H, *J* = 10.8 Hz), 7.21–7.32 (m, 5H, ar).

#### 4.1.7. General Procedure for the Synthesis of **45**–**50**

In a 100 mL flask, equipped with a reflux condenser and a magnetic stirrer, benzyl piperidine **23** or the proper piperazine **24**–**28** (5 mmol), alkyl bromide **44** (5 mmol), K_2_CO_3_ (10 mmol), and MeCN (30 mL) were added. The resulting mixture was refluxed for 14 h. The warm suspension was filtered and the resulting filtrate was concentrated under reduced pressure. The crude material was purified by crystallization.

2-(2-(4-Benzylpiperidin-1-yl)ethyl)isoindoline-1,3-dione (**45**). Yield 54%. Mp: 100–102 °C (acetonitrile). ^1^H-NMR (CDCl_3_): 1.18–1.29 (m, 2H), 1.48–1.54 (m, 1H), 1.60–1.63 (m, 2H), 1.97 (t, 2H, *J* = 10.8 Hz), 2.51–2.52 (m, 2H), 2.61 (t, 2H, *J* = 6.9 Hz), 2.97 (d, 2H, *J* = 11.3 Hz), 3.84 (t, 2H, *J* = 6.9 Hz), 7.13–7.15 (m, 2H, ar), 7.17–7.21 (m, 1H, ar), 7.26–7.30 (m, 2H, ar), 7.72–7.75 (m, 2H, ar), 7.85–7.87 (m, 2H, ar). IR: 1770, 1708, 1705. Anal. calcd. for (C_22_H_24_N_2_O_2_): C, 75.85%; H, 6.94%; N, 8.04%. Anal. found: C, 76.17%; H, 7.23%; N, 8.33%.

2-(2-(4-Phenylpiperazin-1-yl)ethyl)isoindoline-1,3-dione (**46**) [36]. Yield 45%. Mp: 152–154 °C (acetonitrile). ^1^H-NMR (CDCl_3_): 2.69–2.74 (m, 6H), 3.14–3.17 (m, 4H), 3.89 (t, 2H, *J* = 6.5 Hz), 6.85 (t, 1H,ar, *J* = 7.3 Hz), 6.91–6.93 (m, 2H, ar), 7.24–7.26 (m, 2H, ar), 7.72–7.75 (m, 2H, ar), 7.85–7.88 (m, 2H, ar). IR: 1712. Anal. calcd. for (C_20_H_21_N_3_O_2_): C, 71.62%; H, 6.31%; N, 12.53%. Anal. found: C, 71.95%; H, 6.52%; N, 12.88%.

2-(2-(4-Benzylpiperazin-1-yl)ethyl)isoindoline-1,3-dione (**47**) [37]. Yield 43%. Mp: 88–90 °C (acetonitrile). ^1^H-NMR (CDCl_3_): 2.44 (br s, 4H), 2.57 (br s, 4H), 2.66 (t, 2H, *J* = 6.7 Hz), 3.49 (s, 2H), 3.83 (t, 2H, *J* = 6.7 Hz), 7.24–7.33 (m, 5H, ar), 7.72–7.75 (m, 2H, ar), 7.85–7.87 (m, 2H, ar). Anal. calcd. for (C_21_H_23_N_3_O_2_): C, 72.18%; H, 6.63%; N, 12.03%. Anal. found: C, 72.39%; H, 6.50%; N, 12.29%.

2-(2-(4-Phenethylpiperazin-1-yl)ethyl)isoindoline-1,3-dione (**48**). Yield 42%. Mp: 131–133 °C (acetonitrile). ^1^H-NMR (CDCl_3_): 2.52–2.69 (m, 12H), 2.74–2.82 (m, 2H), 3.85 (t, 2H, *J* = 6.7 Hz), 7.19–7.22 (m, 3H, ar), 7.27–7.31 (m, 2H, ar), 7.71–7.75 (m, 2H, ar), 7.84–7.88 (m, 2H, ar). Anal. calcd. for (C_22_H_25_N_3_O_2_): C, 72.70%; H, 6.93%; N, 11.56%. Anal. found: C, 72.81%; H, 6.81%; N, 11.43%.

2-(2-(4-(4-(2-Methoxyethoxy)phenyl)piperazin-1-yl)ethyl)isoindoline-1,3-dione (**49**). Yield 36%. Mp: 124–126 °C (acetonitrile). ^1^H-NMR (CDCl_3_): 2.69–2.74 (m, 6H), 3.04–3.06 (m, 4H), 3.46 (s, 3H), 3.74 (t, 2H, *J* = 4.8 Hz), 3.88 (t, 2H, *J* = 6.6 Hz), 4.08 (t, 2H, *J* = 4.8 Hz), 6.87 (s, 4H, ar), 7.72–7.74 (m, 2H, ar), 7.84–7.87 (m, 2H, ar). IR: 1697. Anal. calcd. for (C_23_H_27_N_3_O_4_): C, 67.46%; H, 6.65%; N, 10.26%. Anal. found: C, 67.55%; H, 7.01%; N, 10.33%.

2-(2-(4-(Furan-2-carbonyl)piperazin-1-yl)ethyl)isoindoline-1,3-dione (**50**). Yield 61%. Mp: 146–148 °C (acetonitrile). ^1^H-NMR (CDCl_3_): 2.57–2.60 (m, 4H), 2.69 (t, 2H, *J* = 6.4 Hz), 3.74 (br s, 4H), 3.85 (t, 2H, *J* = 6.4 Hz), 6.47–6.48 (m, 1H, ar), 6.97–6.98 (m, 1H, ar), 7.48–7.49 (m, 1H, ar), 7.72–7.76 (m, 2H, ar), 7.85–7.89 (m, 2H, ar). Anal. calcd. for (C_19_H_19_N_3_O_4_): C, 64.58%; H, 5.42%; N, 11.89%. Anal. found: C, 64.67%; H, 5.69%; N, 12.17%.

#### 4.1.8. Ethyl 4-(4-(2-(1,3-dioxoisoindolin-2-yl)ethyl)piperazin-1-yl)benzoate (**51**)

In a 100 mL flask, equipped with a reflux condenser and a magnetic stirrer, the aryl piperazine **43** (5 mmol), alkyl bromide **44** (5 mmol), Et_3_N (6 mmol), and MeCN (60 mL) were added. The resulting mixture was refluxed for 24 h. The solution was concentrated under reduced pressure and the oily residue was treated with water (20 mL). The solid was filtered, washed with diethyl ether, and used as such in the next step. Yield 46%. ^1^H-NMR (DMSO-*d*_6_): δ 1.28 (t, 3H, *J* = 7.5 Hz), 2.53–2.65 (m, 6H), 3.22–3.27 (m, 4H), 3.70–3.75 (m, 2H), 4.23 (q, 2H, *J* = 7.5 Hz), 6.96 (d, 2H, ar, *J* = 7.9 Hz), 7.76 (d, 2H, ar, *J* = 7.9 Hz), 7.81–7.92 (m, 4H, ar).

#### 4.1.9. General Procedure for the Synthesis of **58**–**61**

Benzaldehyde (5 mmol) was added to a solution of 4-aminomethylpiperidine **52** (5 mmol) in absolute ethanol (10 mL), and the mixture was heated under reflux for 24 h. After cooling, the solvent was removed by evaporation at reduced pressure. Oily *N*-(piperidin-4-ylmethyl)-1-phenylmethanimine **53 [39]** was thus obtained, and used in a subsequent reaction without further purification. The imine derivative **53** (2.6 mmol) was dissolved in acetone (15 mL) containing potassium carbonate (5.1 mmol) and the proper bromide derivative **54**–**57** (3.1 mmol). The mixture thus obtained was stirred at room temperature for 12 h, then suspension was filtered and the solvent was evaporated at reduced pressure. The remaining oily residue without further purification was used in the next step.

*N*-((1-(2-Methoxyethyl)piperidin-4-yl)-1-phenylmethanimine (**58**). Yield 67%. ^1^H-NMR (DMSO-*d*_6_): 1.04–1.14 (m, 1H), 1.17–1.26 (m, 1H), 1.57–1.67 (m, 4H), 1.89–1.94 (m, 1H), 2.41–2.44 (m, 2H), 2.84–2.93 (m, 2H), 3.23 (s, 3H), 3.38–3.45 (m, 4H), 7.42–7.45 (m, 3H, ar), 7.72–7.74 (m, 2H, ar), 8.30 (s, 1H, CH).

*N*-((1-Benzylpiperidin-4-yl)methyl)-1-phenylmethanimine (**59**) [39]. Yield 90%. ^1^H-NMR (DMSO-*d*_6_): 1.22–1.25 (m, 2H), 1.62–1.65 (m, 3H), 1.91 (t, 2H, *J* = 10.9 Hz), 2.78–2.81 (m, 2H), 3.42–3.45 (m, 4H), 7.18–7.28 (m, 5H, ar), 7.40–7.44 (m, 3H, ar), 7.70–7.72 (m, 2H, ar), 8.30 (s, 1H, CH).

*N*-((1-(4-Methoxybenzyl)piperidin-4-yl)methyl)-1-phenylmethanimine (**60**). Yield 90%. ^1^H-NMR (DMSO-*d*_6_): 1.15–1.27 (m, 2H), 1.59–1.65 (m, 3H), 1.83–1.93 (m, 2H), 2.79–2.81 (m, 2H), 3.38 (s, 2H), 3.45–3.46 (m, 2H), 3.73 (s, 3H), 6.86 (d, 2H, ar, *J* = 8.5 Hz), 7.19 (d, 2H, ar, *J* = 8.5 Hz), 7.41–7.45 (m, 3H, ar), 7.72–7.74 (m, 2H, ar), 8.30 (s, 1H, CH).

*N*-((1-Phenethylpiperidin-4-yl)methyl)-1-phenylmethanimine (**61**) [40]. Yield 90%. ^1^H-NMR (DMSO-*d*_6_): 1.11–1.27 (m, 2H), 1.59–1.73 (m, 3H), 1.94 (t, 2H, *J* = 10.8 Hz), 2.69–2.73 (m, 2H), 3.11–3.14 (m, 2H), 3.45–3.47 (m, 2H), 3.73 (t, 2H, *J* = 7.2 Hz), 7.15–7.33 (m, 5H, ar), 7.44–7.45 (m, 3H, ar), 7.73–7.74 (m, 2H), 8.31 (s, 1H, CH).

### 4.2. Pharmacological Assays

#### 4.2.1. Cell Culture and Membrane Preparation

CHO cells transfected with hA_1_, hA_2A_, hA_2B_, and hA_3_ ARs were grown adherently and maintained in Dulbecco’s modified Eagle’s medium with nutrient mixture F12, containing 10% fetal calf serum, penicillin (100 U/mL), streptomycin (100 μg/mL), L-glutamine (2 mM), geneticin (G418; 0.2 mg/mL) at 37 °C in 5% CO_2_/95% air [44]. For membrane preparation, the cells were washed with phosphate-buffered saline and scraped off T75 flasks in an ice-cold hypotonic buffer (5 mMTris-HCl, 1 mM EDTA, pH 7.4). The cell suspensions were homogenized with a Polytron, centrifuged for 30 min at 40,000× *g* at 4 °C and the resulting membrane pellets were used for competition binding experiments [44].

#### 4.2.2. Competition Binding Experiments

All synthesized compounds have been tested for their affinity to hA_1_, hA_2A_, and hA_3_ ARs. Competition experiments to hA_1_ ARs were performed incubating 1 nM [^3^H]-8-cyclopentyl-1,3-dipropylxanthine ([^3^H]-DPCPX) with membrane suspension (50 μg of protein/100 μL) and different concentrations of the examined compounds at 25 °C for 90 min in 50 mM TrisHCl, pH 7.4. Non-specific binding was defined as binding in the presence of 1 μM DPCPX and was always <10% of the total binding [44]. Inhibition experiments to hA_2A_ ARs were performed incubating 1 nM of [^3^H]-ZM241385 with the membrane suspension (50 μg of protein/100 μL) and different concentrations of the examined compounds for 60 min at 4 °C in 50 mMTris-HCl (pH 7.4), 10 mM MgCl_2_. Non-specific binding was evaluated in the presence of 1 μM ZM241385 and was about 20% of the total binding [45]. Competition binding experiments to A_3_ ARs were carried out incubating the membrane suspension (50 μg of protein/100 μL) with 0.5 nM [^125^I]-*N*^6^-(4-aminobenzyl)-*N*-methylcarboxamidoadenosine **(**[^125^I]-ABMECA) in the presence of different concentrations of the examined compounds for an incubation time of 120 min at 4 °C in 50 mMTris-HCl (pH 7.4), 10 mM MgCl_2_, 1 mM EDTA. Non-specific binding was defined as binding in the presence of 1 μM ABMECA and was always < 10% of the total binding [46]. Bound and free radioactivity were separated by filtering the assay mixture through Whatman GF/B glass fiber filters using a Brandel cell harvester (Brandel Instruments, Unterföhring, Germany). The filter bound radioactivity was counted in a Packard Tri Carb 2810 TR scintillation counter (Perkin Elmer, Waltham, MA, USA).

#### 4.2.3. Cyclic AMP Assays

CHO cells transfected with hAR subtypes were washed with phosphate-buffered saline, detached with trypsin, and centrifuged for 10 min at 200× *g*. Cells were seeded in a 96-well white half-area microplate (Perkin Elmer, Boston, USA) in a stimulation buffer composed of Hank Balanced Salt solution, 5 mM HEPES, 0.5 mM Ro 20-1724, 0.1% BSA, 1 IU/mL adenosine deaminase. cAMP levels were then quantified by using the AlphaScreencAMP detection kit (Perkin Elmer, Waltham, MA, USA) following the manufacturer’s instructions [47]. At the end of the experiments, plates were read with the Perkin Elmer EnSight Multimode Plate Reader.

#### 4.2.4. Data Analysis

The protein concentration was determined according to a Bio-Rad method with bovine albumin as a standard reference. Inhibitory binding constant (Ki) values were calculated from those of IC_50_ according to the Cheng and Prusoff equation Ki = IC_50_/(1 + [C*]/K_D_*), where [C*] is the concentration of the radioligand and K_D_* is its dissociation constant [46]. K_i_ and IC_50_ values were calculated by the non-linear regression analysis using the equation for a sigmoid concentration-response curve (Graph-PAD Prism, San Diego, CA, USA).

## 5. Conclusions

In conclusion, the herein reported structural investigation has led to a good number of new 7-amino-2-(furan-2-yl)-thiazolo[5,4-*d*]pyrimidines, featuring piperidine or piperazine substituents at position 5, endowed with potent and selective hA_2A_ AR inverse agonist activities. Among them, compound 11 bearing a phenylpiperazine-ethylamino chain at position 5, showed the highest hA_2A_ AR binding affinity and potency. Furthermore, the SwissADME prediction indicated that compounds 8, 11, and 19 exhibited good drug-likeness properties.

## Figures and Tables

**Table 1:**
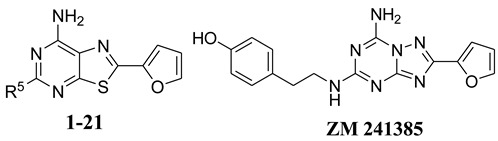
Binding affinities (K_i_) at hA_1_, hA_2A_, and hA_3_ ARs and potencies (IC_50_) at hA_2B_ ARs.

	R_5_	hA_1_AR ^a^ K_i_ (nM) (I%) ^e^	hA_2A_AR ^b^ K_i_ (nM)	hA_2B_AR ^c^ IC_50_ (nM) (I%) ^e^	hA_3_AR ^d^ K_i_ (nM) (I%) ^e^
**1**	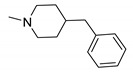	586 ± 37	594 ± 48	>10,000 (21%)	>10,000 (8%)
**2**	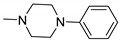	296 ± 22	64 ± 5	>10,000 (15%)	>10,000 (18%)
**3**	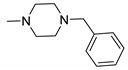	180 ± 15	58 ± 5	10,000 (26%)	868 ± 82
**4**	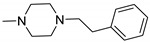	1213 ± 114	237 ± 17	>10,000 (11%)	197 ± 16
**5**	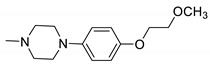	2355 ± 213	326 ± 27	>10,000 (7%)	127 ± 15
**6**	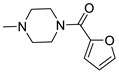	2766 ± 249	137 ± 11	>10,000 (10%)	816 ± 74
**7**	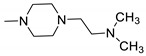	345 ± 28	29 ± 3	>10,000 (23%)	>10,000 (34%)
**8**	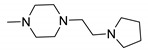	638 ± 56	15.1 ± 1.3	>10,000 (19%)	>10,000 (25%)
**9** ^f^	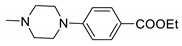	4536 ± 312	279 ± 23	>10,000 (38%)	2679 ± 221
**10**	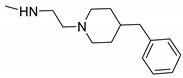	725 ± 67	187 ± 16	>10,000 (22%)	>10,000 (22%)
**11**	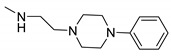	102 ± 9	8.62 ± 0.74	>10,000 (13%)	>10,000 (15%)
**12**	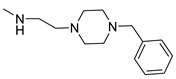	798 ± 72	92 ± 8	>10,000 (26%)	>10,000 (17%)
**13**	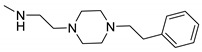	522 ± 47	37 ± 3	>10,000 (29%)	>10,000 (33%)
**14**	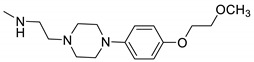	452 ± 38	18.3 ± 1.9	>10,000 (17%)	1492 ± 126
**15**	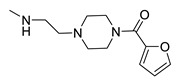	436 ± 36	10.8 ± 1.0	>10,000 (16%)	>10,000 (23%)
**16**	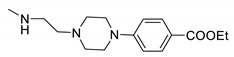	>10,000 (31%)	264 ± 24	>10,000 (26%)	>10,000 (34%)
**17**	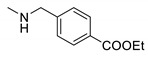	>10,000 (29%)	>10,000 (33%)	>10,000 (17%)	>10,000 (28%)
**18**	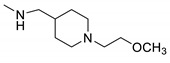	524 ± 41	802 ± 73	>10,000 (11%)	>10,000 (28%)
**19**	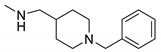	365 ± 29	15.2 ± 1.7	>10,000 (18%)	>10,000 (35%)
**20**	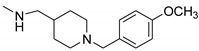	152 ± 11	88 ± 9	>10,000 (24%)	>10,000 (29%)
**21**	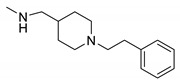	247 ± 18	483 ± 34	>10,000 (27%)	>10,000 (21%)
**ZM 241385**		188 ± 16	0.94 ± 0.07	51 ± 4	672 ± 51

Data are expressed as means ± SEM. Affinity values obtained from the displacement of specific [^3^H]DPCPX ^a^, [^3^H]ZM241383 ^b^, or [^125^I]AB-MECA ^d^ binding to hA_1_ARs, hA_2A_ARs, or A_3_ARs, respectively (*n* = 3–6). ^c^ Potency (IC_50_) in cAMP assays to hA_2B_ARs. ^e^ Percentage of inhibition (I%) is determined at 10 μM concentration of the tested compounds. ^f^ Ref. 31.

**Table 2:** Potency (IC_50_) of selected compounds on cyclic AMP assays in CHO cells expressing hA_2A_ AR.

Compounds	Potency IC_50_, nM	Intrinsic Activity	Pharmacological Behavior
**8**	13.8 ± 1.2	−44 ± 3	Inverse agonist
**11**	7.42 ± 0.68	−52 ± 4	Inverse agonist
**14**	15.2 ± 1.3	−51 ± 5	Inverse agonist
**15**	9.42 ± 0.87	−67 ± 5	Inverse agonist
**19**	14.8 ± 1.4	−64 ± 4	Inverse agonist
**ZM 241385**	1.42 ± 0.11	−48 ± 4	Inverse agonist

Data are expressed as means ± SEM.

## Supplementary Materials

The following are available online at https://www.mdpi.com/1424-8247/13/8/161/s1, Figure S1: Inhibition curves of cAMP levels in hA_2A_ CHO cells by selected compounds in comparison with the reference compound ZM 241385. Table S1: Selected physicochemical and pharmacokinetic properties and drug-likeness predictions of analyzed compounds **8**, **11**, **14**, **15**, **19**.

## References

[1] Borea P.A., Gessi S., Merighi S., Vincenzi F., Varani K. (2018). Pharmacology of adenosine receptors: The state of the art. Physiol. Rev..

[2] Borea P.A., Gessi S., Merighi S., Vincenzi F., Varani K. (2017). Pathological overproduction: The bad side of adenosine. Br. J. Pharmacol..

[3] Al-Attraqchi O.H.A., Attimarad M., Venugopala K.N., Nair A., Al-Attraqchi N.H.A. (2019). Adenosine A_2A_ receptor as a potential drug target—Current status and future perspectives. Curr. Pharm. Des..

[4] Domenici M.R., Ferrante A., Martire A., Chiodi V., Pepponi R., Tebano M.T., Popoli P. (2019). Adenosine A(2A) receptor as potential therapeutic target in neuropsychiatric disorders. Pharmacol Res..

[5] Zheng J., Zhang X., Zhen X. (2019). Development of adenosine A(2A) receptor antagonists for the treatment of Parkinson’s Disease: A recent update and challenge. ACS Chem. Neurosci..

[6] Flor A.M., Moreau J.L., Poli S.M., Riemer C., Steward L. (2005). 4-Hydroxy-4-methyl-piperidine-1-carboxylic acid-(4-methoxy-7-morpholin-4-yl-benzothiazol-2-yl) Amide. US Patent.

[7] Minetti P., Tinti M.O., Carminati P., Castorina M., Di Cesare M.A., Di Serio S., Gallo G., Ghirardi O., Giorgi F., Giorgi L. (2005). 2-n-Butyl-9-methyl-8-[1,2,3]-triazol-2-yl-9H-purin-6-ylamine and analogues as A_2A_ adenosine receptor antagonists. Design, synthesis, and pharmacological characterization. J. Med. Chem..

[8] Gillespie R.J., Bamford S.J., Botting R., Comer M., Denny S., Gaur S., Griffin M., Jordan A.M., Knight A.R., Lerpiniere J. (2009). Antogonists of the human A(2A) adenosine receptor. Design, synthesis, and preclinical evaluation of 7-aryltriazolo[4,5-d]pyrimidines. J. Med. Chem..

[9] Hodgson R.A., Bedard P.J., Varty G.B., Kazdoba T.M., Di Paolo T., Grzelak M.E., Pond A.J., Hadjtahar A., Belanger N., Gregoire L. (2010). Preladenant, a selective A_2A_ receptor antagonist, is active in primate models of movement disorders. Exp. Neurol..

[10] Pinna A. (2014). Adenosine A_2A_ receptor antagonists in Parkinson’s disease: Progress in clinical trials from the newly approved istradefylline to drugs in early development and those already discontinued. CNS Drugs.

[11] Chen J.F., Cunha R.A. (2020). The belated US FDA approval of the adenosine A_2A_ receptor antagonist istradefylline for treatment of Parkinson’s disease. Purinergic Signal..

[12] Dall’Igna O.P., Fett P., Gomes M.G., Souza D.O., Cunha R.A., Lara D.L. (2007). Caffeine and adenosine A_2a_ receptor antagonists β-amyloid (25–35)-induced cognitive deficits in mice. Exp. Neurol..

[13] Congreve M., Brown G.A., Borodovsky A., Lamb M.L. (2018). Targeting adenosine A_2A_ receptor antagonism for treatment of cancer. Expert Opin. Drug Discov..

[14] Vijayan D., Young A., Teng M.W.L., Smyth M.J. (2017). Targeting immunosuppressive adenosine in cancer. Nat. Rev. Cancer.

[15] Ohta A., Gorelik E., Prasad S.J., Ronchese F., Lukashev D., Wong M.K.K., Huang X., Caldwell S., Liu K., Smith P. (2006). A_2A_ adenosine receptor protects tumors from antitumor T cells. Proc. Natl. Acad. Sci. USA.

[16] Merighi S., Battistello E., Giacomelli L., Varani K., Vincenzi F., Borea P.A., Gessi S. (2019). Targeting A_3_ and A_2A_ adenosine receptors in the fight against cancer. Exp. Opin. Ther. Targets.

[17] Merck Sharp and Dohme Corp A phase Ib/II Study to Evaluate the Safety and Tolerability of Preladenant as a Single Agent and in Combination with Pembrolizumab in Subjects with Advanced Malignancies. NCT03099161.

[18] Mediavilla-Verela M., Castro J., Chiappori A., Noyes D., Hernandez D.C., Allard B., Stagg J., Antonia S.J. (2017). A novel antagonist of the immune checkpoint protein adenosine A_2A_ receptor restores tumor infiltrating lymphocyte activity in the context of the tumor microenvironment. Neoplasia.

[19] Corvus Pharmaceuticals, Inc A phase 1/1b, Open Label, Multicenter, Repeat-Dose, Dose-Selection Study of CPI-444 as a Single Agent and in Combination with Atezolizumab in Patients with Selected Incurable Cancers. NCT02655822.

[20] Congreve M., Andrews S.P., Dorè A.S., Hollestenin K., Hurrell E., Langmead C.J., Mason J.S., Ng I.W., Tehan B., Zhukov A. (2012). Discovery of 1,2,4-triazine derivatives as adenosine A_2A_ antagonists using structure based drug design. J. Med. Chem..

[21] Varano F., Catarzi D., Vincenzi F., Betti M., Falsini M., Ravani A., Borea P.A., Colotta V., Varani K. (2016). Design, synthesis and pharmacological characterization of 2-(2-furanyl)thiazolo[5,4-*d*]pyrimidine-5,7-diamine derivatives: New potent A_2A_ adenosine receptor inverse agonists with antinociceptive activity. J. Med. Chem..

[22] Poli D., Falsini M., Varano F., Betti M., Varani K., Vincenzi F., Pugliese A.M., Pedata F., Dal Ben D., Thomas A. (2017). Imidazo[1,2-*a*]pyrazin-8-amine core for the design of new adenosine receptor antagonists: Structural exploration to target the A_3_ and A_2A_ subtypes. Eur. J. Med. Chem..

[23] Squarcialupi L., Betti M., Catarzi D., Varano F., Falsini M., Ravani A., Pasquini S., Vincenzi F., Salmaso V., Sturlese M. (2017). The role of 5-arylalkylamino- and 5-piperazino moieties on the 7-aminopyrazolo[4,3-*d*]pyrimidine core in affecting adenosine A_1_ A_2A_ receptor affinity and selectivity profiles. J. Enzym. Inhib. Med. Chem..

[24] Falsini M., Squarcialupi L., Catarzi D., Varano F., Betti M., Dal Ben D., Marucci G., Buccioni M., Volpini R., De Vita T. (2017). The 1,2,4-triazolo[4,3-*a*]pyrazin-3-one as a versatile scaffold for the design of potent adenosine human receptor antagonists. Structural investigations to target the A_2A_ receptor subtype. J. Med. Chem..

[25] Varano F., Catarzi D., Falsini M., Vincenzi F., Pasquini S., Varani K., Colotta V. (2018). Identification of novel thiazolo[5,4-*d*]pyrimidine derivatives as human A_1_ and A_2A_ adenosine receptor antagonists/inverse agonists. Bioorg. Med. Chem..

[26] Varano F., Catarzi D., Vincenzi F., Falsini M., Pasquini S., Borea P.A., Colotta V., Varani K. (2018). Structure-activity relationship studies and pharmacological ch.; aracterization of N^5^-heteroarylalkyl-substituted-2-(2-furanyl)-thiazolo[5,4-*d*]pyrimidine-5,7-diamine-based derivatives as inverse agonists at human A_2A_ adenosine receptor. Eur. J. Med. Chem..

[27] Varano F., Catarzi D., Falsini M., Dal Ben D., Buccioni M., Marucci G., Volpini R., Colotta V. (2019). Novel human adenosine receptor antagonists based on the 7-amino-thiazolo[5,4-*d*]pyrimidine scaffold. Structural investigations at the 2-, 5- and 7- positions to enhance affinity and tune selectivity. Bioorg. Med. Chem. Lett..

[28] Falsini M., Catarzi D., Varano F., Ceni C., Dal Ben D., Marucci G., Buccioni M., Volpini R., Di Cesare Mannelli L., Lucarini E. (2019). Antioxidant-conjugated 1,2,4-triazolo[4,3-*a*]pyrazin-3-one derivatives: Highly potent and selective human A_2A_ adenosine receptor antagonists possessing protective efficacy in neuropathic pain. J. Med. Chem..

[29] Falsini M., Catarzi D., Varano F., Dal Ben D., Marucci G., Buccioni M., Volpini R., Di Cesare Mannelli L., Ghelardini C., Colotta V. (2019). Novel 8-amino-1,2,4-triazolo[4,3-*a*]pyrazin-3-one derivatives as potent human adenosine A_1_ and A_2A_receptorantagonists. Evaluation of their protective effect against β-amyloid induced neurotoxicity in SHSY5Y cells. Bioorg. Chem..

[30] Falsini M., Ceni C., Catarzi D., Varano F., Dal Ben D., Marucci G., Buccioni M., Navia A.M., Volpini R., Colotta V. (2020). New 8-amino-1,2,4-triazolo[4,3-*a*]pyrazin-3-one derivatives. Evaluation of different moieties on the 6-aryl ring to obtain potent and selective human A_2A_ adenosine receptor antagonists. Bioorg. Med. Chem. Lett..

[31] Varano F., Catarzi D., Vincenzi F., Pasquini S., Pelletier J., Lopes Rangel Fietto J., Espindola Gelsleichter N., Sarlandie M., Guilbaud A., Sevigny J. (2020). Structural investigation on thiazolo[5,4-*d*]pyrimidines to obtain dual-acting blockers of CD73 and adenosine A_2A_ receptor as potential antitumor agents. Bioorg. Med. Chem. Lett..

[32] Shaquiquzzaman M., Verma G., Marella A., Akhter M., Akhtar W., Khan M.F., Tasneem S., Alam M.M. (2015). Piperazine scaffold: A remarkable tool in generation of diverse pharmacological agents. Eur. J. Med. Chem..

[33] Long J.Z., Jin X., Adibekian A., Li W., Cravatt B.F. (2010). Characterization of tunable piperidine and piperazine carbamates as inhibitors of endocannabinoid hydrolases. J. Med. Chem..

[34] Silverman L.S., Caldwell J.P., Greenlee W.J., Kiselgof E., Matasi J.J., Tulshian D.B., Arik L., Foster C., Bertorelli R., Monopoli A. (2007). 3H-[1,2,4]-Triazolo[5,1-*i*]purin-5-amine derivatives as adenosine A_2A_ antagonists. Bioorg. Med. Chem. Lett..

[35] Chun C., Schmitzer A.R. (2011). A pseudorotaxane umbrella thread with chloride transmembrane transport properties. Med. Chem. Commun..

[36] Mejuch T., Garivet G., Hofer W., Kaiser N., Fansa E.K., Ehrt C., Koch O., Baumann M., Ziegler S., Wittinghofer A. (2017). Small-molecule inhibition of the UNC119-cargo interaction. Angew. Chem. Int. Ed..

[37] Piemontese L., Tomas D., Hiremathad A., Capriati V., Candeias E., Cardoso S.M., Chaves S., Santos M.A. (2018). Donezepil structure-based hybrids as potential multifunctional anti-Alzheimer’s drug candidates. J. Enzym. Inhib. Med. Chem..

[38] Kubota D., Ishikawa M., Yamamoto M., Murakami S., Hachisu M., Katano K., Ajito K. (2006). Tricyclic pharmacophore-based molecules as novel integrin α_v_β_3_ antagonists. Part 1: Design and synthesis of a lead compound exhibiting α_v_β_3_/α_IIb_β_3_ dual antagonistic activity. Bioorg. Med. Chem..

[39] Diouf O., Depreux P., Chavatte P., Paupaert J.H. (2000). Synthesis and preliminary pharmacological results on new naphthalene derivatives as 5-HT_4_ receptor ligands. Eur. J. Med. Chem..

[40] Furlotti G., Alisi M.A., Cazzola N., Ceccacci F., Garrone B., Gasperi T., La Bella A., Leonelli F., Loreto M.A., Magarò G. (2018). Targeting serotonin 2A and adrenergic α_1_ receptors for ocular antihypertensive agents: Discovery of 3,4-dihydropyrazino[1,2-b]indazol-1(2H)-one derivatives. ChemMedChem.

[41] Caulkett P.W.R., Jones G., Collis M.G., Poucher S.M. (1991). Preparation of (amino)heteroaryl[1,2,4]triazolo[1,5-a]triazines and Related Compounds as Adenosine A2 Receptor Antagonists. Eur Patent Appl.

[42] Daina A., Michielin O., Zoete V. (2017). SwissADME: A free web tool to evaluate pharmacokinetics, drug-likeness and medicinal chemistry friendliness of small molecules. Sci. Rep..

[43] Daina A., Zoete V. (2016). A BOILED-Egg to predict gastrointestinal absorption and brain penetration of small molecules. ChemMedChem.

[44] Vincenzi F., Targa M., Romagnoli R., Merighi S., Gessi S., Baraldi P.G., Borea P.A., Varani K. (2014). TRR469, a potent A_1_ adenosine receptor allosteric modulator, exhibits anti-nociceptive properties in acute and neuropathic pain models in mice. Neuropharmacology.

[45] Varani K., Massara A., Vincenzi F., Tosi A., Padovan M., Trotta F., Borea P.A. (2009). Normalization of A_2A_ and A_3_ adenosine receptor up-regulation in rheumatoid arthritis patients by treatment with anti-tumor necrosis factor alpha but not methotrexate. Arthritis Rheum..

[46] Varani K., Merighi S., Gessi S., Klotz K.N., Leung E., Baraldi P.G., Cacciari B., Romagnoli R., Spalluto G., Borea P.A. (2000). [^3^H]MRE 3008F20: A novel antagonist radioligand for the pharmacological and biochemical characterization of human A_3_ adenosine receptors. Mol. Pharmacol..

[47] Ravani A., Vincenzi F., Bortoluzzi A., Padovan M., Pasquini S., Gessi S., Merighi S., Borea P.A., Govoni M., Varani K. (2017). Role and function of A_2A_ and A_3_ adenosine receptors in patients with ankylosing spondylitis, psoriatic arthritis and rheumatoid arthritis. Int. J. Mol. Sci..

