# Supplementary material for: Piperazine- and Piperidine-Containing Thiazolo[5,4-d]pyrimidine Derivatives as New Potent and Selective Adenosine A2A Receptor Inverse Agonists
Source: Pharmaceuticals (Basel). 2020 Jul 24;13(8):161. doi: 10.3390/ph13080161 (PMC7465344; doi:10.3390/ph13080161)
Supplement: Supplementary file 1 [file pharmaceuticals-13-00161-s001.pdf]

Supplementary Materials

Piperazine- and piperidine-containing thiazolo[5,4-*d*]pyrimidine derivatives as new potent and selective adenosine A_{2A} receptor inverse agonists.

Flavia Varano[1]*, Daniela Catarzi[1], Erica Vigiani[1], Fabrizio Vincenzi[2], Silvia Pasquini[2], Katia Varani[2], Vittoria Colotta[1].

1 Dipartimento di Neuroscienze, Psicologia, Area del Farmaco e Salute del Bambino, Sezione di Farmaceutica e Nutraceutica, Universita'degli Studi di Firenze, Via Ugo Schiff 6, 50019 Sesto Fiorentino (FI), Italy; vittoria.colotta@unifi.it (V.C.); daniela.catarzi@unifi.it (D.C.); erica.vigiani@unifi.it (E.V.).

2 Dipartimento di Morfologia, Chirurgia e Medicina Sperimentale, Università degli Studi di Ferrara, Via Fossato di Mortara 17-19, 44121 Ferrara, Italy; vrk@unife.it (K.V.); fabrizio.vincenzi@unife.it (F.V.); silvia.pasquini@unife.it (S.P.)

* Correspondence: flavia.varano@unifi.it

Figure S1. Inhibition curves of cAMP levels in hA_{2A} CHO cells by selected compounds in comparison with the reference compound ZM 241385.

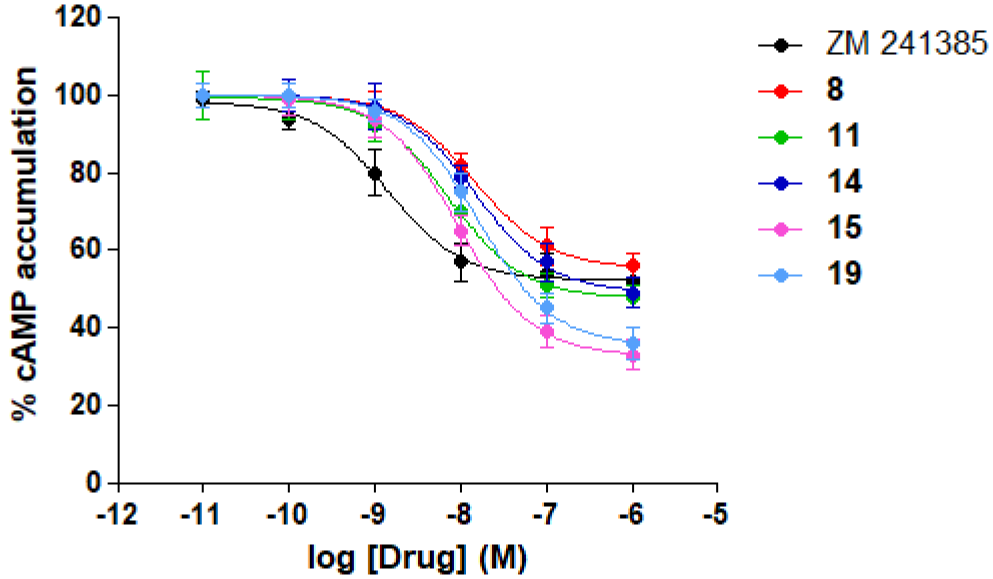

Table S1 Selected physicochemical and pharmacokinetic properties and drug-likeness predictions of analyzed compounds **8**, **11**, **14**, **15**, **19**.

	**8**	**11**	**14**	**15**	**19**
Molecular weight g/mol	399.51	421.52	495.60	439.49	420.53
Heavy atoms	28	30	35	31	30
Aromatic heavy atoms	14	20	20	19	20
Fraction Csp^3	0.53	0.29	0.38	0.30	0.32
Rotatable bonds	5	6	10	7	6
H-bond acceptors	6	5	7	7	5
H-bond donors	1	2	2	2	2
Molar refractivity	122.31	127.21	144.40	124.15	124.65
TPSA [$Å^2$]	115.79	124.58	143.04	154.79	121.34
log $P_{o/w}$ (XLOGP3)	2.20	3.36	3.18	2.09	4.00
Consensus log $P_{o/w}$	1.94	2.56	2.63	1.71	3.34
Log S	-3.74	-4.67	-4.68	-3.87	-5.06
pKa*	5.0	7.7	7.7	7.1	9.3
GI absorption	High	High	Low	Low	High
BBB permeant	No	No	No	No	No
PgP substrate	Yes	Yes	Yes	Yes	Yes
CYP1A2 inhibitor	Yes	Yes	No	No	Yes
CYP2C19 inhibitor	No	Yes	Yes	No	Yes
CYP2C9 inhibitor	Yes	Yes	Yes	Yes	Yes
CYP2D6 inhibitor	Yes	Yes	Yes	Yes	Yes
CYP3A4 inhibitor	Yes	Yes	Yes	Yes	Yes
Log Kp (skin permeation) [cm/s]	-7.18	-6.49	-7.07	-7.50	-6.03
Lipinsky	Yes	Yes	Yes	Yes	Yes
Bioavailability score	0.55	0.55	0.55	0.55	0.55
PAINS	0 alert	0 alert	1 alert	0 alert	0 alert

* calculated with ChemDrawUltra 9.0
